# Hospital Construction

**Published:** 1899-11-11

**Authors:** 


### The Institutional Workshop.

### HOSPITAL CONSTRUCTION.

### THE BELFAST ROYAL VICTORIA HOSPITAL.

#### A Great Experiment.

A great experiment is being tried in the construction of the Royal Victoria Hospital at Belfast, which we will consider more in detail when the building is completed. The general principle, however, on which this hospital has been planned is so entirely opposed to what has been long accepted as the rule in such buildings, that its progress will be watched with very great interest, and all the more so from the fact that the architect, Mr. William Henman, F.R.I.B.A., is well known as an enthusiastic advocate of the plenum system of ventilation. In describing the plans of the new Royal Victoria Hospital to the recent Congress of the Sanitary Institute, Mr. Henman said that the real disadvantage of the pavilion plan, as seen particularly in some large and recently constructed hospitals, was that of administration, in consequence of the great distances which had to be traversed by the staff. He thought that the direction in which advance should now be sought was in the concentration of the wards, and he pointed out that, in view of the fact that in the plenum system the windows were never opened, it was quite possible to build the wards close together side by side. Such an arrangement would secure greater comfort to the patients, would simplify ventilation by mechanical means, would very considerably reduce the length of the corridors, and by dispensing with the inconvenience of staircases and lifts would greatly facilitate administration. This, then, is the principle which Mr. Henman is putting into practice in the new hospital at Belfast. In regard to the administrative block we need say nothing beyond stating that it is a building of four storeys above a basement, and that it contains, among other things, the nurses' home.

The interest lies in the wards. It was a requirement that each of the honorary physicians should have the control of a male and female ward adjoining each other, and that in connection with each group of four medical wards there should be a large class-room and a clinical room. Also that each honorary surgeon should have control of a male and female ward side by side, in connection with which there should be an operating-room, with anæsthetic room adjoining. To meet these conditions eight wards of 16 beds each are provided for medical cases, and the same number for surgical cases; one ward of 16 beds for gynæcological cases; two wards each of eight beds for ophthalmic cases; a large out-patients' department; and a pathological department and lecture theatre, all on the ground level.

The wards and their accessory rooms are placed side by side, with no open space intervening; all are on one floor level, and practically under one roof, and are approached by short corridors, branching off a main corridor running at right angles to the length of the wards. The axis of the wards is almost north and south, and at the south end of each of the 16-bed wards there is a large window opening on to a covered balcony. The wards are lighted from end to end by lantern lights, glazed on the almost vertical sides, and as the buildings will not be overshadowed, sunlight can be admitted at all times according to requirements, draw down blinds being provided for the purpose of regulating the light. Mr. Henman thinks that wards thus lighted will be even more cheerful than if they had windows along the sides, and that they will generally afford greater comfort to the patients.

In connection with every ward of 16 beds there is a single bed ward, a bath-room, lavatory, and conveniences, and also linen and clothes stores. A ward kitchen serves for each pair of wards, except in regard to those devoted to gynæcology, where the department is self-contained, and possesses a separate operating-room. The strong point in this plan is its compactness, and we need hardly say that as an almost necessary corollary of this compactness is the provision of mechanical ventilation. Clearly the plan of the hospital and the character of the ventilation almost of necessity go together. Of course, it would be easy enough to ventilate the wards through the lantern lights. This is a thing that has been done many a time, but when it becomes a question of getting the air to move in the short connecting corridors and the subsidiary rooms, and arranging for the aerial separation of the different parts of the building, it may well be doubted whether it would be possible to maintain such a hospital in a healthy condition except by aid of mechanical ventilation. The success of the plan must, then, depend upon the success of the ventilation, and it is only fair to add that so it is intended by the architect to do. Granting that, there can be no doubt that the plan lends itself admirably to the plenum system of ventilation. The main air-ducts are placed under the corridors, and from them separate flues are carried up to the several wards, rooms, and corridors; the air outlets are at the floor-level, with flues descending to ducts by which the vitiated air passes to the open through ventilating turrets at the ends of the wards. By this arrangement it is said that all ducts and flues will be easily accessible for cleaning; that each ward and room will be practically isolated from every other in consequence of each receiving its own fresh air supply direct; and that no necessity will exist for intercepting lobbies between wards and conveniences because the air from the latter will not be allowed to travel to the former. The air intake is at the east end of the main corridor, and will provide for a supply of cleansed and tempered air equal to a total change of air ten times per hour, night and day, throughout the buildings. Both the fans and engines used for purposes of ventilation will be in duplicate, and will run alternately, in order that one at least may always be available. The architect expresses the hope that the working expenses of this installation will not exceed one-half of that of any installation of the plenum system as yet laid down.

We hope that this great experiment, which, as Mr. Henman admits, "entirely revolutionises preconceived ideas as to what is essential in hospital design," will be successful. We are by no means converts to the necessity for mechanical ventilation where, as in hospitals, a large cubic space has to be allowed to each person for other reasons than those connected merely with ventilation, however desirable the plan may be, and indeed is, where people are more closely crowded together, as in schools and factories. But if it is to be used in hospitals it is only right that the buildings to which it is to be applied should be so constructed as to give it every advantage, and that the architect should be allowed to give free play to his ingenuity in making it a success. We may be quite sure that what can be done will be done by Mr. Henman in this direction.

